# Editorial: Innovative approaches to exercise assessment and prescription in non-communicable diseases

**DOI:** 10.3389/fspor.2025.1560372

**Published:** 2025-01-24

**Authors:** Raphael Martins de Abreu, Beatrice Cairo, Marlus Karsten, Patrick Feiereisen

**Affiliations:** ^1^ Department of Health, LUNEX University of Applied Sciences, Differdange, Luxembourg; ^2^ LUNEX ASBL Luxembourg Health & Sport Sciences Research Institute, Differdange, Luxembourg; ^3^ Department of Biomedical Sciences for Health, University of Milan, Milan, Italy; ^4^ Department of Physiotherapy, Santa Catarina State University, Florianópolis, Santa Catarina, Brazil; ^5^ Department of Cardiology, Centre Hospitalier de Luxembourg, Luxembourg, Luxembourg

**Keywords:** rehabilitation, physical exercise, heart rate, metabolic disease, diabetes mellitus

**Editorial on the Research Topic**
Innovative approaches to exercise assessment and prescription in non-communicable diseases

Non-communicable diseases (NCDs) are a group of medical conditions that are not caused by infectious agents and cannot be transmitted directly from one individual to another. They are typically chronic in nature and progress slowly over time. NCDs are primarily driven by societal factors and lifestyle choices, including inadequate nutrition, tobacco use, excessive alcohol consumption, and insufficient physical activity (1). NCDs represent a major global health issue, contributing to 74% of all deaths globally, according to the World Health Organization (2). Physical exercise plays an important role in addressing the burden of NCDs. However, there is a critical need to explore innovative and non-conventional exercise-based treatment strategies in order to increase individuals adherence to prevention, prehabilitation and rehabilitation programs. In addition, innovative approaches to patients’ assessment should be explored to improve effectiveness of interventions, increase the accuracy of screening, and address the global prevalence of NCDs.

In the study by Romanchuk on “Peculiarities of cardio-respiratory relationships in qualified athletes with different types of heart rhythm regulation according to respiratory maneuver data,” researchers examined the influence of respiratory maneuvers on autonomic regulation of heart rate. The study highlights the role of parasympathetic and sympathetic influences in modulating arterial baroreflex sensitivity under varying breathing rates. By differentiating types of heart rate regulation using a combination of cardiovascular and respiratory indices, this work provides a novel approach to identifying functional overstrain in athletes. These findings have implications for tailoring training regimens, particularly in optimizing respiratory control and improving performance resilience, which could be further investigated for application in other populations and clinical conditions, such as NCDs.

The growing prevalence of type 2 diabetes mellitus (T2DM) requires innovative, non-pharmacological interventions. “Acute and chronic effects of inspiratory muscle training in patients with type 2 diabetes mellitus: A systematic review of randomized controlled trials”, Breuil-Marsal et al. address the impact of inspiratory muscle training (IMT) on cardiac autonomic function, glucose regulation, and exercise capacity. The findings suggest that IMT may be a non-conventional form of exercise to enhance parasympathetic activity, improve glucose variability, and bolstering physical endurance in T2DM patients. Despite methodological differences across studies, IMT emerges as a potential adjunctive therapy. Future investigations should aim to refine protocols and explore the mechanistic pathways linking respiratory muscle training to metabolic improvements.

A framework for resistance exercise training is increasingly recognized for its therapeutic role in oncology, as detailed in “A Practical Framework for the Design of Resistance Exercise Interventions in Oncology Research Settings.” This narrative review by Fairman, provides an approach for overcoming the logistical and methodological complexities of designing resistance exercise interventions for patients diagnosed with cancer. The guidance provided by this article is invaluable for researchers seeking to tailor exercise interventions to the multifaceted needs of cancer survivors, ultimately improving quality of life and mitigating treatment-related side effects.

In “Factors Associated with Lower Quarter Performance-Based Balance and Strength Tests: A Cross-Sectional Analysis from the Project Baseline Health Study”, the authors explore the demographic, health, and behavioral factors influencing balance and strength testing in adults living independently in the community. In this study, Taylor et al., identified significant associations between performance measures and variables such as sex, race, education level, and specific health conditions such as non-alcoholic fatty liver disease. By affirming the utility of tests such as the single-legged balance test and sit-rise test as prognostic tools, this research supports their broader use in preventive care. The findings also highlight the importance of stratified analyses by age and health status to refine performance benchmarks.

With the integration of machine learning (ML) into healthcare and research, the potential for data-driven decision-making has expanded exponentially. “Machine learning models for assessing risk factors affecting health care costs: 12-month exercise-based cardiac rehabilitation” by Hautala et al., uses ML to identify key predictors of healthcare costs during cardiac rehabilitation. Diabetes emerged as the most significant cost driver, followed by body mass index and systolic blood pressure. These findings underline the value of ML in enhancing the accuracy of risk stratification and resource allocation. By identifying modifiable risk factors, this study demonstrates how ML can inform personalized interventions and optimize the economic efficiency of rehabilitation programs.

Early identification of cardiometabolic risk in young adults is critical to curbing the progression of metabolic syndrome (MetS). “Exploring Gait Velocity as a Predictor of Cardiometabolic Disease Risk in Young Adults” by Thorsen et al., examines gait speed and related parameters as potential surrogates for MetS risk. The study reveals a strong inverse relationship between gait speed and a continuous cardiometabolic risk score, the MetSindex. These findings expand the application of gait analysis from older to younger populations, providing a non-invasive, accessible tool for early risk detection. By focusing on preventive strategies, this research supports the integration of functional movement assessment into routine health monitoring.

The articles in this Research Topic reflect a shared commitment to advancing our understanding of the complex interplay between physical function, cardiometabolic health, and intervention strategies. Importantly, they emphasize the need for tailored approaches that take into account individual variability. While these studies represent significant progress, they also highlight areas for future investigation. The heterogeneity of methodologies, sample populations, and intervention protocols underscores the need for standardization and replication. In addition, as digital health tools and ML technologies are increasingly integrated into clinical and research settings, ethical considerations related to data privacy and equitable access must be prioritized. The contributions to this special issue illuminate critical pathways for improving cardiometabolic health and advancing clinical exercise physiology research. By addressing both the acute and chronic dimensions of health, these studies lay the foundation for innovative and effective interventions to manage NCDs.
